# Predicting and optimizing reactive oxygen species metabolism in *Punica granatum* L. through machine learning: role of exogenous GABA on antioxidant enzyme activity under drought and salinity stress

**DOI:** 10.1186/s12870-024-04740-2

**Published:** 2024-01-23

**Authors:** Saeedeh Zarbakhsh, Ali Reza Shahsavar, Ali Afaghi, Mirza Hasanuzzaman

**Affiliations:** 1 Department of Horticultural Science, Faculty of Agriculture, Shiraz University, Shiraz, Iran; 2 Faculty of Electrical and Computer Engineering, University of Tabriz, Tabriz, Iran; 3 Department of Agronomy, Faculty of Agriculture, Bangla Agricultural University, Sher-ESher-E-Bangla Nagar, Dhaka, 1207 Bangladesh

**Keywords:** Hydrogen peroxide, Machine learning, Malondialdehyde, Peroxidase, Optimization tool, Protein, γ-aminobutyric acid

## Abstract

**Background:**

Drought and salinity stress have been proposed as the main environmental factors threatening food security, as they adversely affect crops' agricultural productivity. As a potential solution, the application of plant growth regulators to enhance drought and salinity tolerance has gained considerable attention. γ-aminobutyric acid (GABA) is a four-carbon non-protein amino acid that accumulates in plants as a response to stressful conditions. This study focused on a comparative assessment of several machine learning (ML) regression models, including radial basis function, generalized regression neural network (GRNN), random forest (RF), and support vector regression (SVR) to develop predictive models for assessing the effect of different concentrations of GABA (0, 10, 20, and 40 mM) on various physio-biochemical traits during periods of drought, salinity, and combined stress conditions. The physio-biochemical traits included antioxidant enzyme activities (superoxide dismutase, SOD; peroxidase, POD; catalase, CAT; and ascorbate peroxidase, APX), protein content, malondialdehyde (MDA) levels, and hydrogen peroxide (H_2_O_2_) levels. The non‑dominated sorting genetic algorithm‑II (NSGA‑II) was employed for optimizing the superior prediction model.

**Results:**

The GRNN model outperformed the other ML algorithms and was therefore selected for optimization by NSGA-II. The GRNN-NSGA-II model revealed that treatment with GABA at concentrations of 20.90 mM and 20.54 mM, under combined drought and salinity stress conditions at 20.86 and 20.72 days post-treatment, respectively, could result in the maximum values for protein content (by 0.80 and 0.69), APX activity (by 50.63 and 51.51), SOD activity (by 0.54 and 0.53), POD activity (by 1.53 and 1.72), CAT activity (by 4.42 and 5.66), as well as lower MDA levels (by 0.12 and 0.15) and H_2_O_2_ levels (by 0.44 and 0.55), respectively, in the ‘Atabaki’ and ‘Rabab’ cultivars.

**Conclusions:**

This study demonstrates that the GRNN-NSGA-II model, as an advanced ML algorithm with a strong predictive ability for outcomes in combined stressful environmental conditions, provides valuable insights into the significant factors influencing such multifactorial processes.

**Supplementary Information:**

The online version contains supplementary material available at 10.1186/s12870-024-04740-2.

## Background

Plant responses to individual stresses have been extensively studied [1–3]. However, in natural environments, plants must cope with multiple simultaneous abiotic stresses [4]. Soil salinity and drought are two particularly important abiotic stresses that often occur together and have a more severe impact on global crop productivity compared to each stress alone [4, 5]. Soil salinity is primarily caused by neutral salts like NaCl and Na_2_SO_4_, with NaCl being the most prevalent [6]. When plants are exposed to drought and salinity stress, one of their physiological reactions is the production of reactive oxygen species (ROS). At high concentrations, ROS can cause damage to various cellular components such as proteins, cell membranes, and nucleic acids (DNA and RNA) [7, 8]. Plant cellular compartments such as chloroplasts, mitochondria, and peroxisomes generate various types of ROS [9]. Hydrogen peroxide (H_2_O_2_) is the most stable type of ROS, making it one of the most studied ROS. Plants have developed various strategies to counteract the harmful effects of ROS and stress-induced damage. One such strategy is the antioxidant defense system, which includes enzymatic and non-enzymatic antioxidants that help maintain redox homeostasis, scavenge ROS, and alleviate stress damage [10]. Some important antioxidant enzymes involved in the defense against ROS include superoxide dismutase (SOD), peroxidase (POD), ascorbate peroxidase (APX), and catalase (CAT). SOD, present in various plant cellular compartments, serves as the initial enzyme, converting superoxide into H_2_O_2_ and oxygen [11]. CAT, APX, and POD contribute to the conversion of H_2_O_2_ into water, providing protection against oxidative damage [12]. Under stressful conditions, the family of isoenzymes known as APX functions as a ROS scavenger, showing a specific affinity for peroxide substrate [13, 14]. It is also involved in the ascorbate–glutathione cycle (ASA-GSH), which safeguards plants by scavenging harmful ROS [15]. While plants have natural defense mechanisms, they may not be sufficient to cope with the combined effects of drought and salinity. Recent studies have shown that the exogenous application of plant growth regulators or bio-stimulants can play a crucial role in improving plant physiological responses to stress. For example, γ-aminobutyric acid (GABA), a non-protein amino acid, has been found as a signaling molecule and a metabolite in plants to regulate defense responses to various abiotic and biotic stresses [16]. The exogenous application of GABA has been shown to enhance plant growth, alleviate oxidative damage caused by stress, and improve stress tolerance in different crop species by scavenging ROS and increasing antioxidant enzyme activities [2, 17, 18]. GABA treatment has been particularly effective in protecting crops from oxidative damage caused by salinity or water deficit in various studies involving *Trifolium repens* cv. Haifa [2], Zea mays [19], *Helianthus annuus* L. [20], and *Phaseolus vulgaris* L. [21].

Pomegranate (*Punica granatum* L.) is a prominent subtropical fruit commonly cultivated in horticulture, believed to have originated in Iran and the Himalayas in northern India [22]. In the entire world, Iran has the largest pomegranate production, cultivar diversity, and quality. However, Iran's agrosystem has faced substantial challenges in recent years due to severe droughts, substandard water resources, and soil salinity [1]. These adverse conditions have had a severe impact on pomegranate crop production, primarily due to the plant's heightened vulnerability to abiotic stressors prevalent in tropical and subtropical regions, such as drought and salinity. The combined effects of drought and salinity stress significantly compromise the yield of pomegranate plants. Consequently, pomegranate serves as an ideal candidate for investigating the protective effects of exogenous GABA against the harsh conditions of water deficit and salinity stress.

The response of plants to stress is highly complex, influenced by multiple factors and their interactions, poses challenges for traditional statistical analysis methods. Traditional statistical techniques like linear regression and variance analysis are more suitable for analyzing small datasets with limited dimensions and are inappropriate for inferring the nonlinear and complex relations in biological systems [23, 24]. Moreover, these techniques are prone to data over-fitting. In recent years, machine learning (ML) algorithms have emerged as a cutting-edge computational tools for analyzing complex, non-linear, high-dimensional, and non-deterministic datasets in various fields, including plant science [25, 26]. Deep learning (DL), a subset of ML, utilizes hierarchical representations and complex nonlinear functions trained from previous layers to automatically learn from data [27]. Convolutional neural networks (CNN), deep neural networks (DNN), and long-short term memory (LSTM) are state-of-the-art DL architectures widely used in remote sensing, crop disease prediction, and plant variety classification [28–30]. ML algorithms such as artificial neural networks (ANNs), support vector machines (SVMs), and random forest (RF) have been successfully used to overcome the challenges posed by non-linear datasets [26, 31]. These computer-based technologies utilize all spectral datasets to address multicollinearity in multiple linear regression models [31]. Radial basis function (RBF), and generalized regression neural network (GRNN) are two most well-known ANN types that have been widely and effectively applied in plant science [32, 33]. The primary advantage of ANNs is their ability to learn, adapt, and generalize to changing experimental conditions, allowing the models to be used with new data [34]. They can also perform non-linear multiple regression [34, 35]. The RF algorithm is a supervised algorithm that can be used for both classification and regression tasks. It is an ensemble method based on decision trees, where multiple dense trees are constructed using bootstrapped training data samples [36]. The use of the bootstrap aggregation method in the RF model helps to reduce the variability in the prediction model [37]. In addition to ANN and RF, SVM has been developed as another approach for data modeling, offering solutions to clustering, classification, and regression problems [38]. SVMs often employ a large number of learning problem formulations to solve quadratic optimization problems, resulting in the SVM training producing results that are at the global optimum [32, 39]. While these models have excellent learning capabilities, they lack interpretability. Therefore, it is necessary to link optimization techniques with mathematical models to interpret the results and determine the significant effects of independent variables on dependent variables. In this context, the genetic algorithm (GA) is an evolutionary single-objective optimization algorithm that is considered one of the effective ML approaches for achieving the best results for a given objective. This method is inspired by Charles Darwin's concepts of "survival of the fittest" and "natural selection" [40]. However, for interpreting multi-objective problems, evolutionary multi-objective optimization algorithms are more suitable. One of the most common used algorithms is the non-dominated sorting genetic algorithm-II (NSGA-II), which can simultaneously optimize several conflicting fitness functions and generate multiple alternative solutions in a single run [41]. Despite the considerable potential of ML techniques in optimizing and predicting plant responses to various abiotic and biotic stresses, their utilization in this field remains relatively limited. Although there are only a few studies available on the implementation of ML techniques for modeling and optimizing plant-based physio-biochemical traits, successful applications have been documented in specific cases. For instance, ML techniques have been effectively employed to optimize the phenolic profile of *Vitis vinifera* [25], extract metabolites from *Capsicum annuum* [42], and evaluate the antioxidant and antimicrobial activity of *Cucumis metuliferus* pulp, skin, and seed [43]. These reports highlight the promising outcomes achieved through the integration of ML techniques in the realm of plant science, paving the way for further exploration and potential advancements in the field.

The purpose of this study is to establish a ML-based method to find the optimal studied parameters associated with the physio-biochemical responses of pomegranate plants subjected to salinity and drought stress. The key highlights of this study encompass the following aspects: (1) examining how two pomegranate cultivars respond to different concentrations of GABA treatment, drought stress, salinity stress, and combined stress conditions in terms of their physio-biochemical responses; (2) comparing the performance of commonly used ML algorithms such as RBF, GRNN, RF, and SVR; and (3) determining the most accurate and efficient ML algorithm and linking it with NSGA-II to predict optimal physio-biochemical parameters based on the optimal parameters studied. In summary, this study makes the following novel contributions:

• Comparing the appropriateness of RBF, GRNN, RF, and SVR nonlinear methods for modeling the effects of GABA treatment, during periods of drought, salinity, and combined stress conditions on oxidative stress parameters, antioxidant enzyme activities, and protein content of pomegranate.
• Identifying the optimal experimental variables to optimize the antioxidant enzyme activities, protein content, and oxidative stress parameters through optimizing the developed model using NSGA-II.

To the best of our knowledge, this study represents the first successful application of ML algorithms to predict the physio-biochemical responses of plants under drought-salinity stress.

## Materials and methods

### Plant material and GABA treatment 

This study was performed on two-year-old pomegranate plants (*Punica granatum* cv. ‘Rabab’ and ‘Atabaki’) which were cultured in 10L black plastic pots with soil and leaf litter (3:2 w/w) and kept in a greenhouse with a temperature of 28 ± 1 °C, relative humidity of 60 ± 5%, and a photoperiod of 16/8 h (light/dark). Every four days, plants were fertilized using ½ Hoagland’s nutrient solution. After four months, plants were subjected to the following stress treatments for 45 days: (i) control (untreated); (ii) moderate drought stress (D; 60% of field capacity); (iii) moderate salt stress (S; 60 mM of NaCl); and (iv) combined drought and salinity stress (D × S; 60% of field capacity and 60 mM of NaCl). To evaluate the effects of GABA treatment, plants were sprayed with different concentrations of GABA (0, 10, 20, and 40 mM) three times at 15-day intervals and immediately exposed to stress treatments. Samples of fully developed leaves, with four biological replicates, were harvested after 14 d, 30 d, and 45 d of stress exposure, respectively, and directly frozen in liquid nitrogen and stored at -80 °C for further analysis.

### The measurement of leaf oxidative damage 

**Fig. 1:**
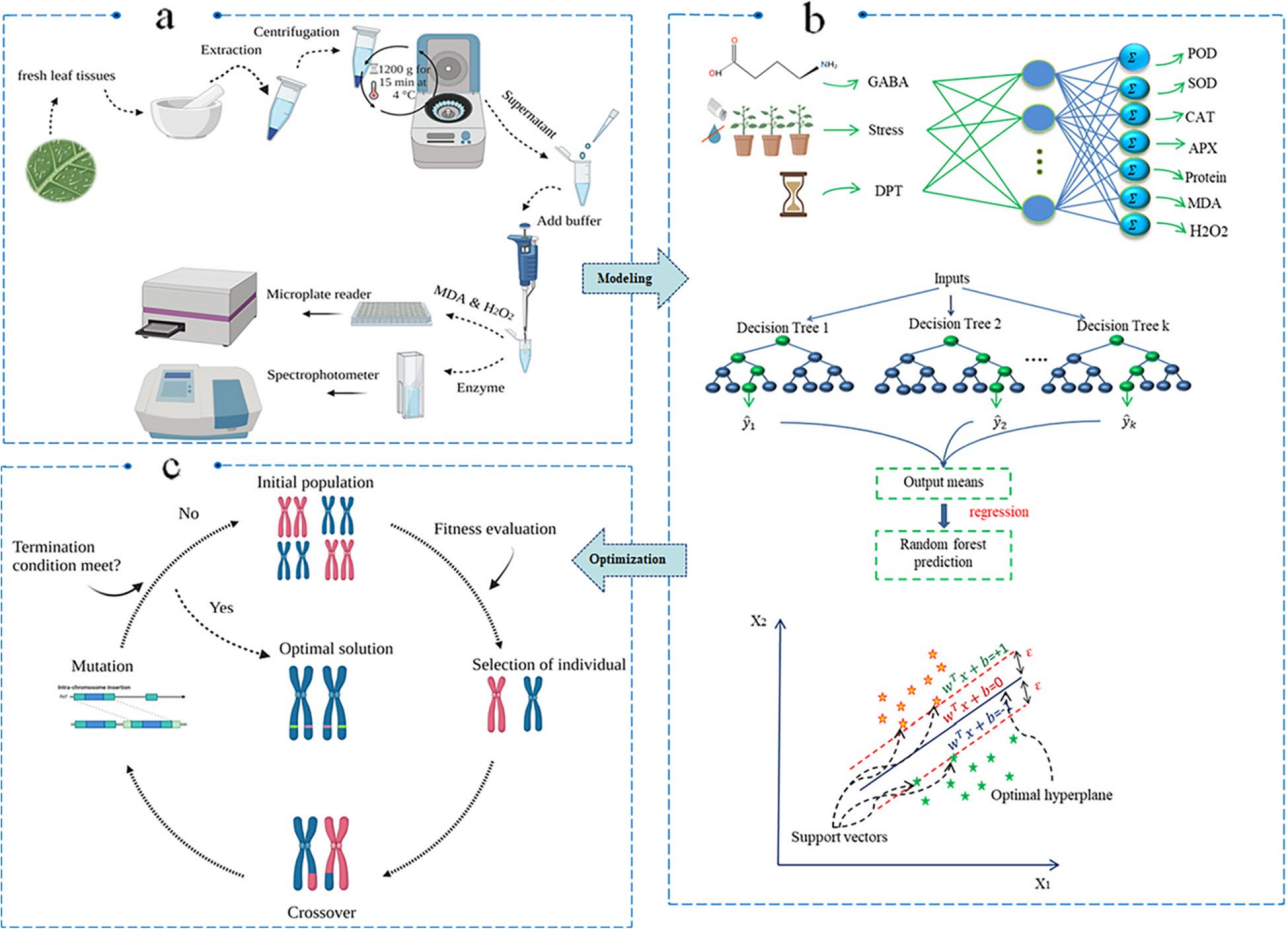
Schematic diagram of the step-by-step methodology used in this study including (**a**) measurement of physiochemical traits, **b** data modeling through artificial neural networks (ANN), random forest (RF), and support vector regression (SVR), and (**c**) main steps of optimization process of physiochemical traits through non-dominated sorting genetic algorithm-II (NSGA-II)

To prepare for the extraction of malondialdehyde (MDA) and hydrogen peroxide (H_2_O_2_) (Fig. 1a), fresh frozen leaves (0.5 g) were homogenized in 5 mL of extraction buffer with 1% trichloroacetic acid (TCA), and then, the resulting homogenate extract was centrifuged for 10 min at 12,000 rpm and 4 °C. The obtained supernatant was used for further analysis.

Lipid peroxidation or MDA content was quantified according to Bai et al. [44] with some modifications. Around 0.25 mL of supernatant was added to a 1 mL reaction mixture consisting of 20% (w/v) TCA and 0.5% (w/v) TBA, and the mixture was boiled in a water bath at 95 °C for 30 min. Subsequently, the mixture was immediately cooled in an ice bath. The supernatant absorbance was measured at 450, 532, and 600 nm using an Epoch Microplate Spectrophotometer (BioTek Instruments, Inc., USA) and expressed on a µmol per g FW basis.

The generation of H_2_O_2_ was determined according to the method of potassium iodide (KI) [45]. The reaction mixture in a total volume of 1 mL included 250 µl of supernatant, 250 µl of K-phosphate buffer (100 mM, pH 7.0), and 500 μl of KI (1 M). The oxidation product was measured at λ = 390 nm and the results were expressed as H_2_O_2_ in mg per g FW.

### Assessment of antioxidant enzyme activities and protein content 

To prepare the extraction of antioxidant enzymes (SOD, POD, CAT, and APX) and total soluble protein (Fig. 1a), fresh leaf tissues (0.5 g) were homogenized with 5 mL of 50 mM K-phosphate buffer (pH 7.0) containing 1% polyvinylpyrrolidone and 0.2 mM ethylenediamine-tetraacetic acid (EDTA). The mixture was centrifuged at 12,000 g for 15 min at 4 °C, and then the acquired supernatant was used to determine the activities of the antioxidant enzymes and soluble proteins.

Superoxide dismutase (SOD) activity was determined by adding 50 μl of enzyme extraction to 950 μl SOD reaction solution (50 mM phosphate buffer, 75 μM nitroblue tetrazolium (NBT), 13 mM L-methionine, 2 μM riboflavin, and 0.1 mM EDTA-Na_2_). The enzyme mixture was placed for 15 min in 4000 lx light; however, the blank was placed in the dark. The absorbance of enzyme solution and blank was compared at 560 nm by using a spectrophotometer (JENWAY-7315, Staffordshire, UK) [46]. The results were expressed as units per mg FW.

Peroxidase (POD) activity was measured according to the protocol described by Chance and Maehly [47]: 20 μl of enzyme extract was mixed with 2.8 mL of POD reaction solution (13 mM guaiacol, 5 mM H_2_O_2_, and 50 mM pH 7.0 phosphate buffer). The absorbance values were determined in terms of oxidized µM of guaiacol spectrophotometrically at 470 nm for 3 min. The results were expressed as units per mg FW.

Catalase (CAT) activity determination was carried out as a decrease in absorbance at λ = 240 nm for 1 min following the disappearance of H_2_O_2_ by the method of Dhindsa et al. [48]. The reaction mixture contained 50 mM phosphate buffer (pH 7.0), 15 mM H_2_O_2_, and 15 μl of enzyme extract. The results were expressed as units per mg FW.

The activity of ascorbate peroxidase (APX) was performed following the procedure developed by Nakano and Asada [49] at an absorbance of 290 nm for 1 min. Briefly, 50 μl enzyme solution was mixed with 0.5 mM ASA, 1.2 mM H_2_O_2_, 0.1 mM EDTA, and 50 mM sodium phosphate buffer (pH 7.0), and the absorption changes of enzyme activity were calculated as units per mg FW.

The amount of soluble protein was determined by following the method of Bradford [50] by using bovine serum albumin as the standard curve and expressed as mg per g of FW. An aliquot of supernatant (20 μl) was analyzed using Coomassie Brilliant Blue-G250 solution as dye, and the absorbance readings were taken at λ = 595 nm. As a result, protein content was expressed as mg per g FW.

### Statistical analysis and modeling tools 

To determine significant differences between the independent and dependent variables, an analysis of variance (one-way ANOVA) was carried out based on a completely randomized design (CRD) with the factorial arrangement using SAS software (version 9.4; SAS Institute, Cary, NC). The correlation between dependent variables was performed using Pearson correlation in the *corrplot* package of R software version 4.3.1. A principal component analysis (PCA-biplot) based on a correlation matrix was created using Minitab version 16 statistical software.

In the current study, the four most well-known ML algorithms were utilized to build the predicting models: radial basis function (RBF), generalized regression neural network (GRNN), RF, and SVR (Fig. 1b). The factors studied or experimental variables (GABA exogenous treatment, stress treatment, and days post-treatment (DPT)) were determined as inputs, and physio-biochemical responses of pomegranate (SOD, POD, APX, CAT, protein, H_2_O_2_, and MDA) were determined as outputs in two pomegranate cultivars (‘Rabab’ and ‘Atabaki’) (Fig. 1b). Prior to using modeling tools, in order to prevent the influence and dominance of a particular dataset over prediction outputs, datasets were standardized by the z-score normalization technique Eq. (1). A regression task typically involves training and testing sets comprising some data samples. Based on trial and error, 85 percent and 15 percent of 192 datasets of each pomegranate cultivar were randomly used in the training and test steps of each modeling technique, respectively. Several biological studies have confirmed the effectiveness of machine learning algorithms in modeling datasets similar to the dataset used in this research such as banana fruit yield with 108 datasets [51], callus growth and development in *Cannabis sativa* with 132 datasets [52], *Juglans regia* L. proliferation with 215 datasets [53], and gene transformation in *Nicotiana tabacum* with 246 datasets [54]. To find the best ML models, the hyperparameters were estimated using a grid or randomized search technique. All ML models' prediction performance was evaluated using K-fold cross-validation procedure [53, 55]. This technique divides the training data into k equal-sized folds. One fold is used as a validation set and the rest as a training set. The model is trained and tested on each fold. The average performance on the k validation sets estimates the model’s skill on new data. This technique reduces the model’s variance and prevents over-fitting or under-fitting [56]. MATLAB software v2020b as a statistical computational tool was used to design the structure of modeling algorithms and optimization processes.

1 $${\text{X}}_{\text{(i)}}^{\text{j}}\text{=}\frac{{\text{X}}_{\text{i}}^{\text{j}}-{\upmu}^{\text{j}}}{{\upsigma}^{\text{j}}}$$

where ${\text{X}}_{\text{i}}^{\text{j}}$, ${\upmu}^{\text{j}}$, and ${\upsigma}^{\text{j}}$ refer to the ${\text{i}}^{\text{th}}$ instance of the ${\text{j}}^{\text{th}}$ value, the mean and standard deviation of the ${\text{j}}^{\text{th}}$ value.

### Radial basis function

The structure of neurons and layers of RBF is like the multilayer perceptron model. In a typical RBF, there are three layers: an input layer, one hidden layer with the non-linear radial basis transfer function, and a linear output layer. Commonly, the Gaussian function (${\varphi }_{i}\left(x\right)$) is located in the hidden layer as a transfer (activation) function (Eq. 2).

2 $${\upvarphi}_{\text{i}}\left({\text{x}}\right)= \text{e} \left(-\frac{\Vert \text{x} - {\text{c}}_{\text{i}}\Vert}{{\upsigma}_{\text{i}}^{2}}\right)$$

where ${\text{x}}$ represents the input vector; ${\text{c}}_{\text{i}}$ and ${\upsigma}_{\text{i}}$ are RBF function center and positive real number, respectively.

3 $$\widehat{\text{Y}}\text{=}\sum_{\text{i=1}}^{\text{n}}{\upvarphi}_{\text{i}}{{\text{w}}}_{\text{i}}$$

where ${\text{w}}_{\text{i}}$ denotes output layer weight, and $n$ is the number of hidden neurons.

### Generalized regression neural network

The GRNN model is a regression-based neural network and a RBF-ANN variant [57]. It learns from a single-pass network and has higher accuracy and speed than back propagation ANN. It uses arbitrary function approximation between input and output layers and predicts the output from the training data. The algorithm has four layers (input, pattern, summation, and output). The input layer receives the input vector and passes it to the pattern layer. The pattern layer connects to two neurons in the summation layer: S and D neurons. They compute the weighted and unweighted sum of the pattern neuron responses, respectively. The GRNN algorithm uses normalized Gaussian kernels and linear activation functions in the hidden and output layer, respectively. The output set is normalized through the summation and output layer. The output of GRNN is calculated by Eqs. (4) and (5).

4 $${\text{D}}_{\text{i}}^{2}\text{=}{\left(\text{X} - {\text{X}}_{\text{i}}\right)}^{\text{T}}\left(\text{X} - {\text{X}}_{\text{i}}\right)$$

5 $${\widehat{\text{Y}}}_{\text{i}}\text{=}\frac{{\sum }_{\text{i=1}}^{\text{N}}{{\text{Y}}}_{\text{i}}{{\text{e}}}^{\left({}^{{\text{-D}}_{\text{i}}^{2}}\!\left/ \!{}_{{2\upsigma}^{2}}\right.\right)}}{{\sum }_{\text{i=1}}^{\text{N}}{{\text{e}}}^{\left({}^{{\text{-D}}_{\text{i}}^{2}}\!\left/ \!{}^{{2\upsigma}^{2}}\right.\right)}}$$

where ${\text{D}}_{\text{i}}^{2}$ represents the Gaussian function between any ${\text{X}}_{\text{i}}$ and ${\text{Y}}_{\text{i}}$ observed data,${\widehat{\text{Y}}}_{\text{i}}$ represents the average of all the weighted output data, ${\text{Y}}_{\text{i}}$ shows the ${\text{i}}^{\text{th}}$ output variable and ${\upsigma}$ is the width parameter.

### Random forest regression

The RF algorithm is a non-parametric ensemble ML technique for classification and regression and is widely used in scientific fields [58]. This tree-based ML model creates multiple decision trees (ntree) from the independent variables using the “bootstrap” or “bagging” method (randomly selected from approximately 70% of the training samples) to combine them into a single model. Also, about one-third of the observations in the learning set are not used in the model construction out-of-bag (OOB) to assess the RF model’s prediction performance. RF does not need a separate set to evaluate the model because of the bagging and OOB approaches [36]. Some advantages of the RF algorithm are that it is less prone to over-fitting, robust to outliers and noise, and free of data distribution assumptions [59, 60]. RF, by combining different independent predictors, can avoid the problem of over-fitting [61]. For optimal model prediction, different parameters (node size, mtry, and ntree) of the model building were set as shown in Eq. (6).

6 $$\text{i.}\widehat{\text{y}}\left({\text{x}}_{\text{i}}\right)\text{=}\frac{1}{{\text{K}}}{\sum }_{\text{k=1}}^{\text{K}}{{\text{T}}}_{{\text{D}}\left({\uptheta}_{\text{k}}\right)}\left({\text{x}}_{\text{i}}\right)$$

where ${\text{x}}_{\text{i}}$ represents the value of the sample proportion, ${\text{D}}\left({\uptheta}_{\text{k}}\right)$ is a different bootstrapped sample, and ${\text{K}}$ is the number of each tree (${\text{T}}_{{\text{D}}\left({\uptheta}_{\text{k}}\right)}$) (k = 1, 2, … K).

### Support vector regression

The SVM is a powerful ML method with a theoretical root [62] that was first developed for classification problems, i.e., SVC, and then extended to regression problems—SVR [59]. Here, we briefly describe the basic idea of SVR that we used in this study. In the training set, each data instance has some attributes or features and one ‘‘target value’’ (class labels). The kernel function was used to map the original input into the feature space. The linear function fit of the kernel-based SVR is given by Eq. (7).

7 $${\text{f}}\left(\text{x,w}\right){\text{=w}}^{\text{T}}\text{x+b}$$

where ${\text{f}}$, ${\text{b}}$, and ${\text{w}}$ determine output value, bias, and weight for the ${\text{i}}^{\text{th}}$ sample point, respectively. Eventually, ${\text{w}}$ and ${\text{b}}$ coefficients will be determined in an optimization process:

8 $$\text{Min=R}\left({\text{C}}\right)\text{=}\frac{1}{{2}}{\Vert {\text{w}}\Vert }^{2}+ \text{C} \frac{1}{{\text{l}}}\sum_{\text{i=1}}^{\text{l}}{{\text{L}}}_{ }\left({\text{y}}_{\text{i}}{\text{,f}}_{\text{i}}\left(\text{x,w}\right)\right)$$

9 $${\left|\text{y-f(x,w)}\right|}_{\upepsilon}\text{=}\left\{\begin{array}{cc}{0} & \left|\text{y-f(x,w)}\right|\le\upepsilon\\ \left|\text{y-f(x,w)}\right|-{\upepsilon} & \text{ Otherwisw}\end{array}\right.$$

where ${\text{L}}_{\upepsilon}$, ${\text{C}}$, and ${\upepsilon}$ (epsilon) represents insensitive loss function (e), the trade-off between model complexity and training error, and an acceptable error (insensitive tube), respectively. The following equation is employed to determine Lagrange multipliers for the dual function of the problem:

10 $$\text{Max} = {\text{L}}_{\textrm{p}}\left({\text{a}}_{\textrm{i}}-{\text{a}}_{\textrm{i}}^{*}\right)\text{=-}\frac{1}{{2}}\sum_{\text{i,j=1}}^{\textrm{l}}\left({\text{a}}_{\textrm{i}}-{\text{a}}_{\textrm{i}}^{*}\right)\left({\text{a}}_{\textrm{j}}-{\text{a}}_{\textrm{j}}^{*}\right){\text{k}}\left({\text{x}}_{\textrm{i}}{,}{\textrm{x}}_{\textrm{j}}\right){-\upepsilon}\sum_{\text{i=1}}^{\text{l}}\left({\text{a}}_{\textrm{i}}{\textrm{+a}}_{\text{i}}^{*}\right)\text{+}\sum_{\text{i=1}}^{\textrm{l}}\left({\text{a}}_{\textrm{i}}-{\text{a}}_{\textrm{i}}^{*}\right){\text{y}}_{\textrm{i}}$$

where ${\text{k}}\left({\text{x}}_{\text{i}}{,}{ \, {\text{x}}}_{\text{j}}\right)$ represents kernel function, which ${\text{x}}_{\text{i}}$ and ${\text{x}}_{\text{j}}$ are each input vectors. Subjected to:

11 $$\left\{\begin{array}{c}\sum\limits_{\text{i=1}}^{\text{l}}\left({\text{a}}_{\text{i}}-{\text{a}}_{\text{i}}^{*}\right)= \text{0} \\ {0}{\le}{\text{a}}_{\text{i}}{\le}{\text{C}}{,} \, \, \, {\text{i=}}{1}{,}{...}{,}{\text{l}}\\ {0}{\le}{\text{a}}_{\text{i}}^{*}{\le}{\text{C,}} \, \, \, {\text{i=}}{1}{,}{...}{,}{\text{l}}\end{array}\right.$$

${\text{b}}$ and ${\text{w}}$ are weight and a bias calculated by minimizing the risk function. The supporting vector is a set of Lagrange multipliers with non-zero grades. Then, SVR is determined as follows:

12 $${\text{f}}\left(\text{x,w}\right){\text{=w}}_{0}^{\text{T}}\text{x+b=}\sum_{\text{i=1}}^{\text{l}}\left({\text{a}}_{\text{i}}-{\text{a}}_{\text{i}}^{*}\right){\text{k}}\left({\text{x,x}}_{\text{i}}\right)+ \text{b}$$

The parameters were initialized as: (${\text{C}}$, ${\upepsilon}$, and ${\text{k}}$) and the radial basis function (RBF) was employed as the kernel function.

### Evaluation of model accuracy and performance 

In the present study, a comparison of the performance and accuracy of the predicted ML models was evaluated via the regression coefficient (R^2^) and the error indicators including root mean square error (RMSE) and mean bias error (MBE, or bias).

13 $${\text{R}}^{2}\text{=1-}\left(\frac{\sum_{\text{i=1}}^{\text{n}}{\left({\text{Y}}_{{\text{e}}{\text{st}}}-{\text{Y}}_{\text{obs}}\right)}^{2}}{\sum_{\text{i=1}}^{\text{n}}{\left({\text{Y}}_{\text{obs}}-\stackrel{\mathrm{-}}{\text{Y}}\right)}^{2}}\right)$$

14 $$\text{RMSE} = \sqrt{\frac{\sum_{\text{i=1}}^{\text{n}}{\left({\text{Y}}_{\text{est}}-{\text{Y}}_{\text{obs}}\right)}^{2}}{\text{n}}}$$

15 $$\text{MBE} = \frac{1}{{\text{n}}}{\sum }_{\text{i=1}}^{\text{n}}\left({\text{Y}}_{\text{est}}-{\text{Y}}_{\text{obs}}\right)$$

where ${\text{Y}}_{\text{obs}}$ and ${\text{Y}}_{\text{est}}$ display the observed values and the estimated values, respectively.

### Multi-objective optimization process via NSGA-II

The best ML algorithm was introduced to the non-dominated sorting genetic algorithm (NSGA-II) to achieve the optimal values of inputs GABA exogenous, stress treatment, DPT, and predict the optimal values of outputs SOD, POD, APX, CAT, Protein, H_2_O_2_, and MDA (Fig. 1c). In this regard, several parameters of the NSGA-II algorithm for the problem were assessed for the best outcome (non-dominated solutions). First, an individual population was created-encoded as chromosomes-that represents the variables of the optimization problem to be solved. Second, elite populations were selected by a tournament selection operator to create a new population using the crossover and mutation method. Also, the crossover function was considered based on the two-point crossover. According to the computational analysis, a good balance between solution quality and computational efficiency can be achieved by setting the NSGA-II parameters. In the current study, the total crossover rate, initial population size, the maximum number of generations, and mutation rate were, respectively, considered as 0.8, 50, 200, and 0.05 in the ‘Atabaki’ cultivar and 0.8, 50, 200, and 0.01 in the ‘Rabab’ cultivar. These values were determined through a trial-and-error process.

### Validation experiments 

In the lab, the predicted input variables obtained from GRNN-NSGA-II were experimentally evaluated to approve the reliability and efficiency of the utilized model. The obtained validation experiment results were compared with predicted results with four biological replicates based on a completely randomized design.

## Results

### Effects of exogenous GABA and drought-salinity stress on pomegranate physio-biochemical response 

The ANOVA results demonstrated that the each of the four factors studied (cultivar, exogenous GABA, stress treatment, and DPT) affected the plant’s physio-biochemical traits (Table S1). Based on the results, the plants’ response to the stress conditions was higher than in the control (non-stress) conditions. In this regard, in two pomegranate cultivars, drought and salinity stress increased the activities of antioxidant enzymes (APX, SOD, POD, and CAT) and protein content, but more so in drought-salinity stress. Also, oxidative stress parameters (MDA and H_2_O_2_ contents) increased quickly in leaves when exposed to stress conditions (Table S1).

**Fig. 2:**
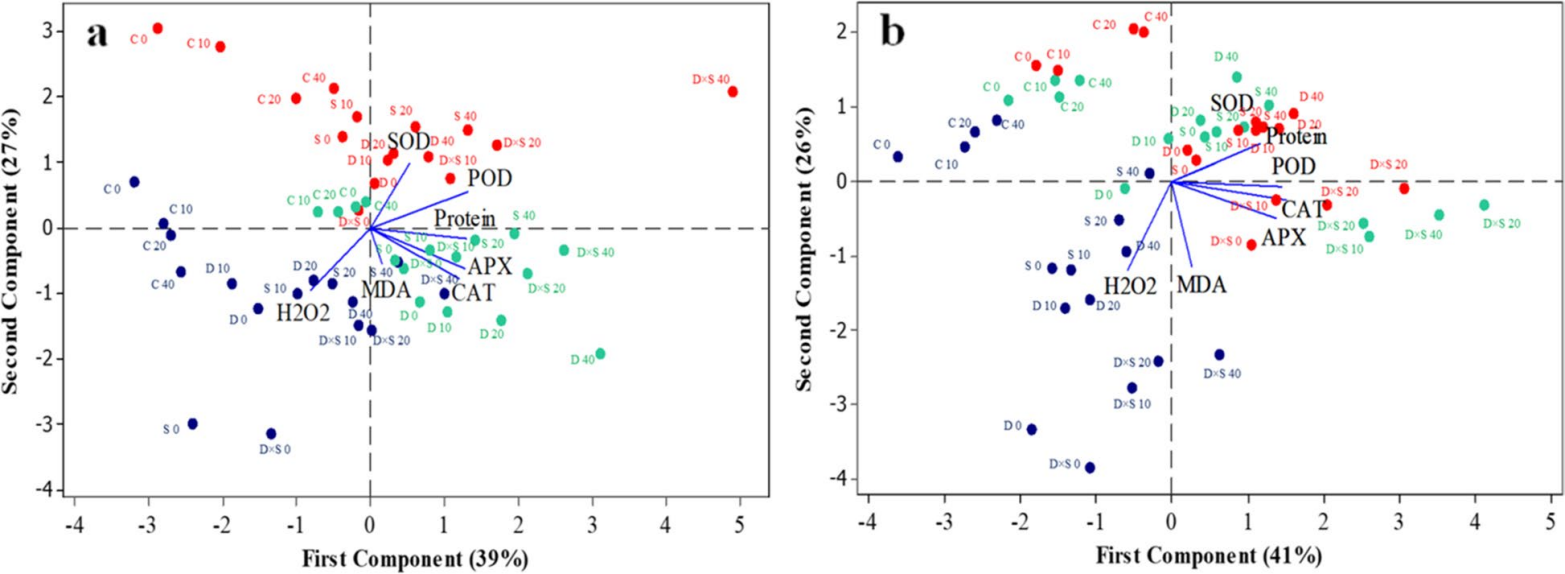
Principal component analysis (PCA) for the pomegranate physio-biochemical properties under studied treatments (**a**) ‘Atabaki’ (**b**) ‘Rabab’. Sample signature: stress treatments are represented as control (C), drought (D), salinity (S), and drought-salinity (D × S). Also, different concentrations of GABA treatment represented as 0, 10, 20, and 40 mM. Blue, red, and green color indicates the 14, 30, and 45 DPT, respectively

**Fig. 3:**
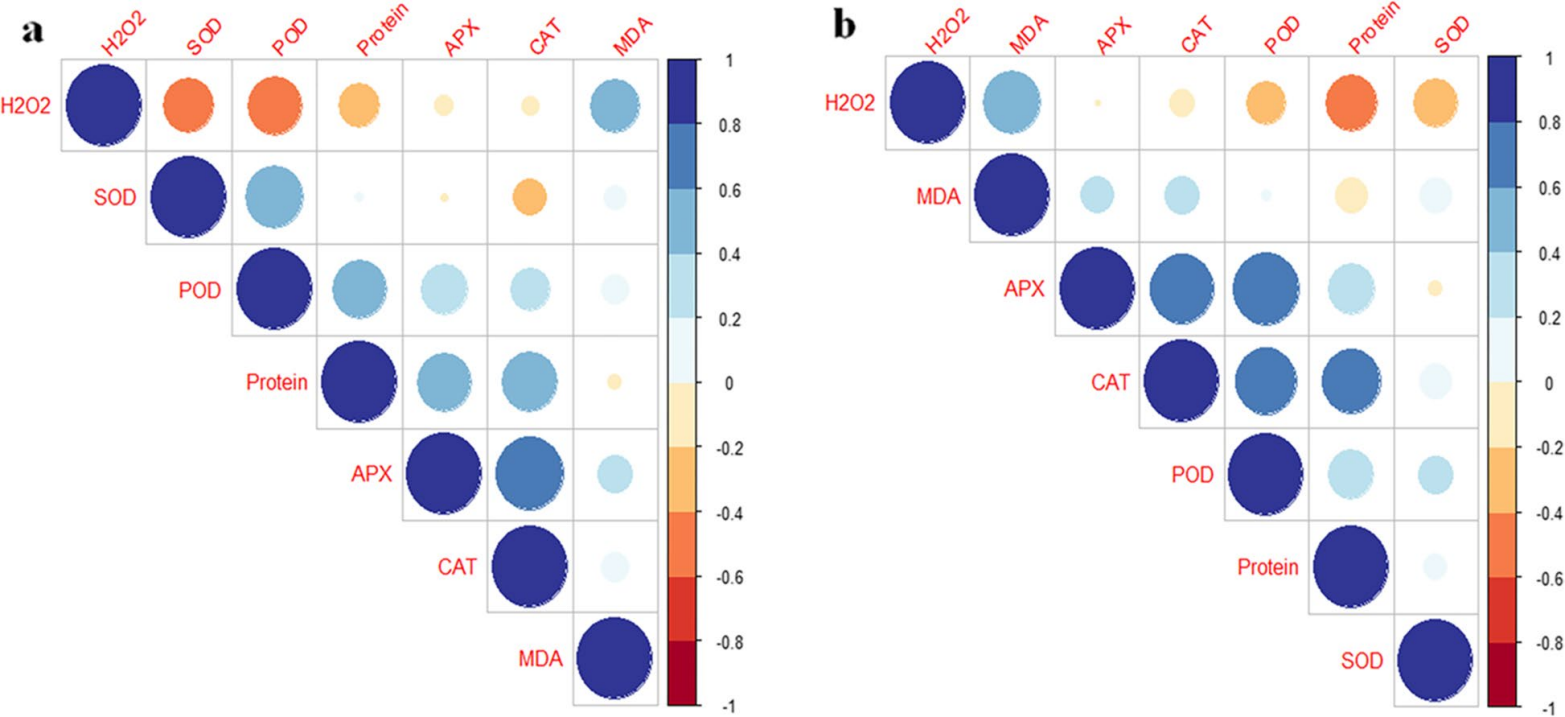
Pearson correlation analysis of physio-biochemical parameters of (**a**) ‘Atabaki’ and (**b**) ‘Rabab’ cultivars in response to studied parameters

In all samples, APX, SOD, POD, CAT, and protein content first exhibited an increasing trend among 14 and 30 DPT plants and then considerably decreased on the last day of stress treatment (45 DPT); however, the values of APX, SOD, POD, CAT, protein, and MDA at 45 DPT were higher than at 14 DPT. In contrast, MDA and H_2_O_2_ concentrations in the leaves of both pomegranate cultivars exhibited the opposite trend and decreased significantly as the stress treatments progressed. However, a slight decrease in the values of activities of antioxidant enzymes, i.e., APX and SOD of the ‘Rabab’ cultivar leaves at 45 DPT under drought stress was observed over those plants at 14 DPT. Also, in the ‘Rabab’ cultivar, similar changes were obtained in SOD activity under salinity stress, control treatment, and drought-salinity stress at 45 DPT in comparison with 14 DPT. However, although antioxidant enzyme activities and oxidative stress parameters in leaves had apparent variations across different experimental DPT, it is noticeable that GABA-treated samples that were exposed to drought and salinity stress showed the highest activities of antioxidant enzymes; whereas, GABA treatment significantly reduced oxidative-relative traits (MDA and H_2_O_2_ contents) at all experimental periods. The significant changes in the investigated parameters were mainly caused by increasing the concentration of GABA. In both cultivars, the plants that received GABA treatment and combined drought-salinity exhibited higher antioxidant enzyme activity and lower oxidative stress than those that received drought and salt stress alone. Under normal (non-stress) conditions, exogenous GABA has no significant impact on investigated parameters, except for POD, CAT, and H_2_O_2_, which were elevated after GABA treatment (P < 0.05). Furthermore, observed changes in investigated values of leaves in the untreated and GABA-treated plants in control (non-stress) or stress conditions were relatively similar in both cultivars (‘Atabaki’ and ‘Rabab’). To better detect the differences between the GABA treatments, cultivars, drought and salinity stress, and DPT, PCA-biplot analysis was calculated among all the physio-biochemical parameters of both cultivars. The first two principal components (PCs) of the ‘Atabaki’ cultivar explained 66% (39% and 27%, respectively) and in the ‘Rabab’ cultivar explained 67% (41% and 26%, respectively) of the total variance in the seven variables space (Fig. 2). The first PC axis of both (‘Atabaki’ and ‘Rabab’) cultivars was related in one extreme (positive values) with high values of traits (protein, CAT, POD, and APX), which is confirmed based on Pearson coefficients of correlation analysis (Figs. 2a, b, 3a, and b). The opposite extreme of the first PC (negative values) was related to the H_2_O_2_ trait. The second PC was illustrated mainly by variations in the protein and SOD. Furthermore, in the ‘Atabaki’ cultivar, particularly in GABA treatment conditions, the 14th DPT showed a close relationship with H_2_O_2_ content, the 30th DPT showed a higher association with SOD and POD, and the 45th DPT displayed a higher association with protein, CAT, APX, and MDA as compared to the other DPT. The ‘Rabab’ cultivar, particularly in GABA treatment conditions, showed higher values of H_2_O_2_ on the 14th DPT, higher values of APX, CAT, POD, protein, and SOD on the 30th DPT, and an intermediate association with values of SOD, APX, and CAT on the 45th DPT (Fig. 2a, b). In both cultivars, a positive correlation between H_2_O_2_ and MDA was observed, as well as a negative correlation among the H_2_O_2_, SOD, and POD, and among the H_2_O_2_ and protein, which were observed in the ‘Atabaki’ and ‘Rabab’ cultivars, respectively (Fig. 3).

### Determination of optimal prediction model 

**Fig. 4:**
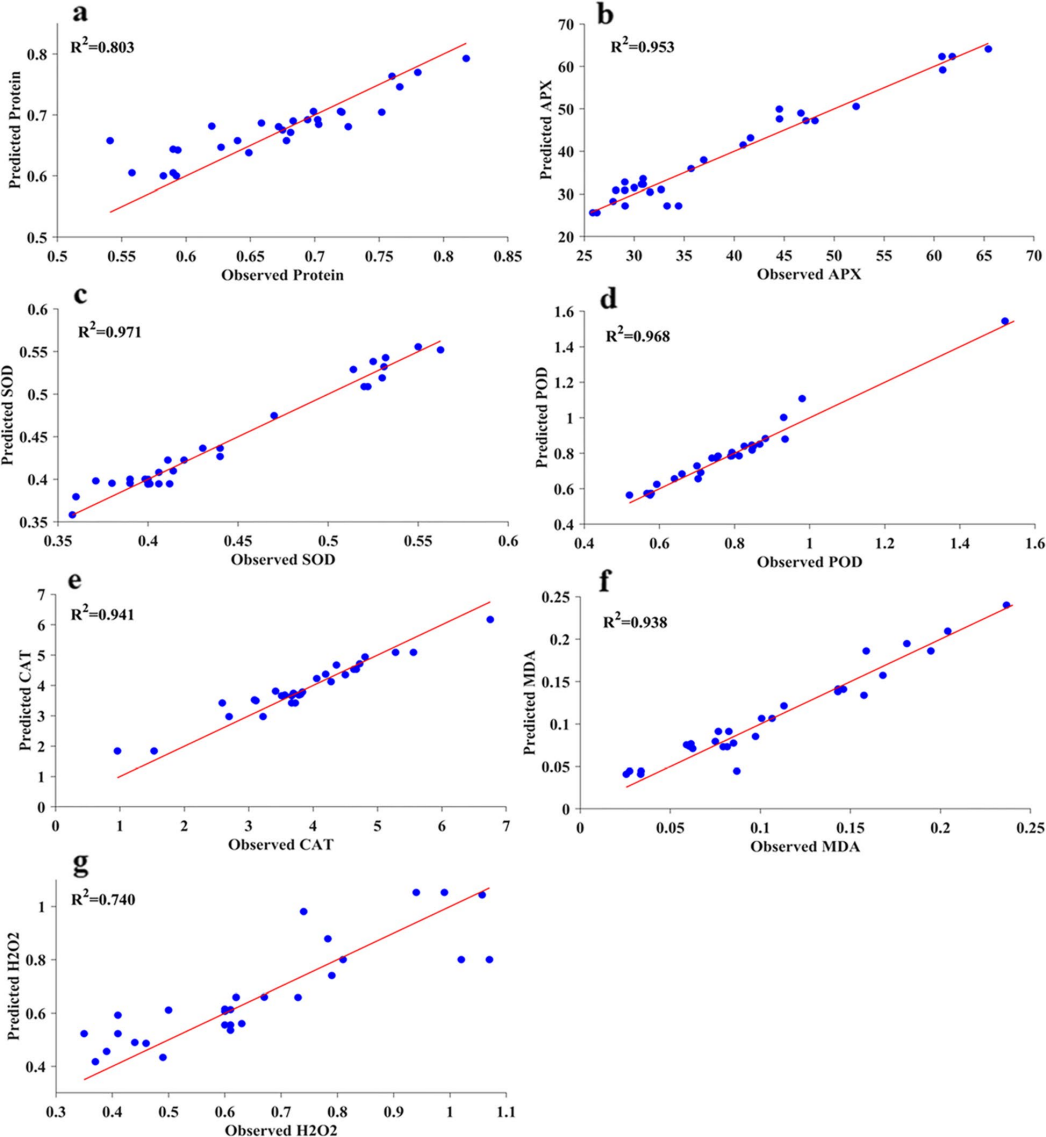
The scatter plot of observed values versus estimated values of (**a**) protein content, **b** ascorbate peroxidase (APX), **c** superoxide dismutase (SOD), **d** peroxidase (POD), **e** catalase (CAT), **f** malondialdehyde (MDA), and **g** hydrogen peroxide (H_2_O_2_) derived from generalized regression neural network model (GRNN) model in ‘Atabaki’ cultivar

**Fig. 5:**
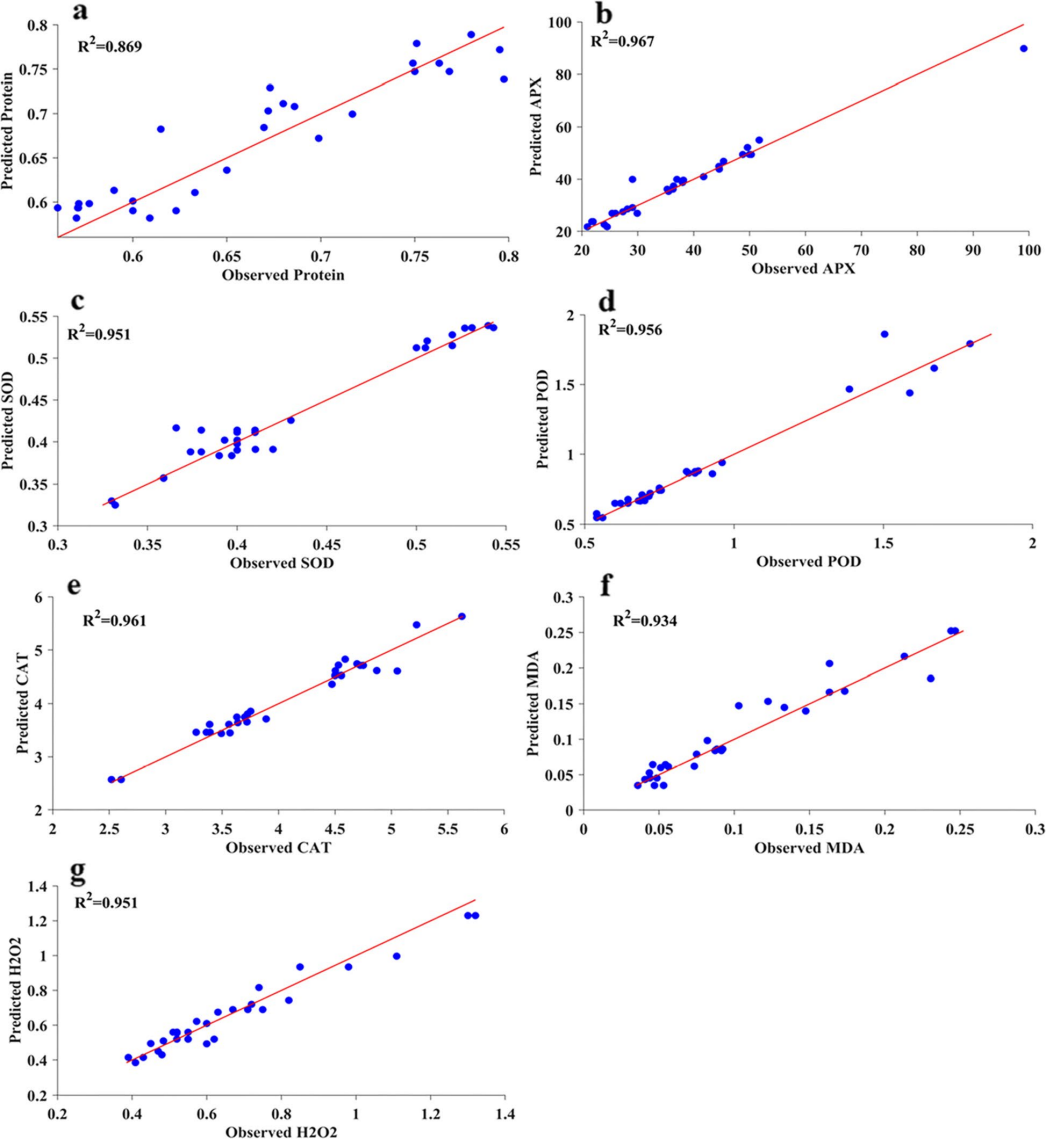
The scatter plot of observed values versus estimated values of (**a**) protein content, **b** ascorbate peroxidase (APX), **c** superoxide dismutase (SOD), **d** peroxidase (POD), **e** catalase (CAT), **f** malondialdehyde (MDA), and **g** hydrogen peroxide (H_2_O_2_) derived from generalized regression neural network model (GRNN) model in ‘Rabab’ cultivar

In this study, we employed several ML algorithms (RBF, GRNN, RF, and SVR) to model and estimate pomegranate physio-biochemical responses. With the help of residual analysis (RMSE and MBE) and a variable R^2^ value, we could select the best-performing model with more confidence. GRNN prediction results for the test subset are shown in Figs. 4 and 5. Also, statistical indicators of all models that were utilized to show the performance of the predictive models are given in Table 1. Results exhibited a high R^2^ value with low RMSE and MBE values for all models. However, the R^2^ values of protein and H_2_O_2_ are relatively lower than the other outputs, indicating the low compatibility between inputs and outputs of protein and H_2_O_2_. In general, comparative analysis of models (Table 1) showed very small differences between models for output variables. Although the R^2^ of SVR, RF, and RBF for estimating some physio-biochemical parameters was higher than that of GRNN, their RMSE and MBE values were lower. Considering the R^2^ and accuracy of model prediction, the GRNN algorithm performed better in estimating output datasets than the other ML algorithms. All the training and test set R^2^ values of the ‘Atabaki’ and ‘Rabab’ cultivars in the GRNN model were above 0.679 and 0.845, respectively, which indicates good performance and high predictability. So, the GRNN algorithm was the best-performing regression model over all other models. With respect to Table 1, calculated R^2^ revealed the order of GRNN, RF, and SVR vs. RBF models were: 0.803, 0.780, and 0.736 vs. 0.608 for protein content of ‘Atabaki’; 0.869, 0.907, and 0.900 vs. 0.856 for protein content of ‘Rabab’; 0.953, 0.962, and 0.953 vs. 0.945 for APX of ‘Atabaki’; 0.967, 0.965, and 0.966 vs. 0.966 for APX of ‘Rabab’; 0.971, 0.967, and 0.966 vs. 0.970 for SOD of ‘Atabaki’; 0.951, 0.963, and 0.947 vs. 0.950 for SOD of ‘Rabab’; 0.968, 0.970, and 0.958 vs. 0.920 for POD of ‘Atabaki’; 0.956, 0.954, and 0.954 vs. 0.952 for POD of ‘Rabab’; 0.941, 0.862, and 0.926 vs. 0.900 for CAT of ‘Atabaki’; 0.961, 0.965, and 0.965 vs. 0.946 for CAT of ‘Rabab’; 0.938, 0.929, and 0.930 vs. 0.913 for MDA of ‘Atabaki’; 0.934, 0.923, and 0.921 vs. 0.919 for MDA of ‘Rabab’; 0.740, 0.616, and 0.757 vs. 0.723 for H_2_O_2_ of ‘Atabaki’; 0.951, 0.918, and 0.931 vs. 0.896 for H_2_O_2_ of ‘Rabab’. Moreover, in both pomegranate cultivars, the regression lines of coefficient (R^2^) have confirmed a strong correlation between the real observed and predicted values of the GRNN for all physio-biochemical parameters (Figs. 4 and 5).

**Table 1:** Performance Comparison of ML algorithms

Cultivar	Subset	Model	Performance metrics	Protein	APX	SOD	POD	CAT	MDA	H_2_O_2_
‘Atabaki’	Training	RBF	R^2^	0.704	0.934	0.978	0.917	0.931	0.906	0.918
			RMSE	0.026	2.574	0.010	0.045	0.213	0.015	0.077
			MBE	-2.207	0.0003	4.277	-0.800	8.271	-14.391	-31.558
		GRNN	R^2^	0.827	0.981	0.981	0.980	0.958	0.950	0.957
			RMSE	0.020	1.378	0.010	0.022	0.167	0.011	0.052
			MBE	0.0002	-21.160	0.0001	9.554	-0.0004	-17.986	-7.805
		RF	R^2^	0.762	0.970	0.981	0.973	0.917	0.942	0.892
			RMSE	0.024	1.754	0.009	0.026	0.242	0.012	0.118
			MBE	-0.0002	-0.052	18.270	0.0007	-0.009	0.0002	-0.004
		SVR	R^2^	0.713	0.980	0.971	0.958	0.941	0.925	0.908
			RMSE	0.025	1.377	0.011	0.028	0.184	0.014	0.083
			MBE	0.002	0.012	0.0004	0.001	0.018	-0.001	0.001
	Testing	RBF	R^2^	0.763	0.945	0.970	0.920	0.900	0.913	0.723
			RMSE	0.043	2.753	0.011	0.054	0.372	0.017	0.131
			MBE	0.004	0.246	0.002	0.008	0.101	0.002	0.038
		GRNN	R^2^	0.803	0.953	0.971	0.968	0.941	0.938	0.740
			RMSE	0.034	2.585	0.011	0.036	0.335	0.014	0.107
			MBE	0.007	0.269	0.002	0.009	0.063	0.002	0.016
		RF	R^2^	0.780	0.962	0.967	0.970	0.862	0.929	0.616
			RMSE	0.036	2.308	0.012	0.034	0.461	0.015	0.134
			MBE	0.002	0.538	0.0005	0.002	0.084	0.002	0.038
		SVR	R^2^	0.736	0.953	0.966	0.958	0.926	0.930	0.757
			RMSE	0.037	2.581	0.012	0.038	0.379	0.015	0.107
			MBE	0.009	0.239	0.002	0.004	0.099	0.004	0.024
‘Rabab’	Training	RBF	R^2^	0.909	0.991	0.973	0.985	0.958	0.941	0.925
			RMSE	0.021	1.480	0.011	0.043	0.127	0.013	0.063
			MBE	-1.781	-0.0005	-1.746	8.967	1.150	-29.518	-1.153
		GRNN	R^2^	0.955	0.995	0.987	0.993	0.972	0.946	0.962
			RMSE	0.016	1.160	0.007	0.027	0.102	0.013	0.045
			MBE	0.0004	0.010	0.870	0.000	-0.0003	19.038	-20.110
		RF	R^2^	0.930	0.994	0.977	0.992	0.966	0.944	0.955
**Cultivar**	**Subset**	**Model**	**Performance metrics**	**Protein**	**APX**	**SOD**	**POD**	**CAT**	**MDA**	**H**_**2**_**O**_**2**_
			RMSE	0.018	1.229	0.010	0.031	0.116	0.013	0.049
			MBE	-0.0007	-0.009	-26.472	-0.0008	0.0006	-0.0003	-0.0012
		SVR	R^2^	0.937	0.993	0.982	0.990	0.959	0.924	0.951
			RMSE	0.018	1.38	0.009	0.039	0.124	0.015	0.052
			MBE	0.002	0.112	-10.158	-0.005	-0.011	-23.775	0.005
	Testing	RBF	R^2^	0.856	0.966	0.950	0.952	0.946	0.919	0.896
			RMSE	0.030	2.970	0.015	0.079	0.175	0.019	0.088
			MBE	0.007	0.120	0.002	0.005	0.017	0.005	-0.019
		GRNN	R^2^	0.869	0.967	0.951	0.956	0.961	0.934	0.951
			RMSE	0.028	3.032	0.015	0.078	0.149	0.017	0.056
			MBE	0.006	0.590	0.004	0.011	0.020	0.004	-0.010
		RF	R^2^	0.907	0.965	0.963	0.954	0.965	0.923	0.918
			RMSE	0.025	3.025	0.013	0.079	0.143	0.018	0.073
			MBE	0.008	0.415	0.004	0.009	0.024	0.002	-0.008
		SVR	R^2^	0.900	0.966	0.947	0.954	0.965	0.921	0.931
			RMSE	0.025	2.990	0.015	0.075	0.142	0.019	0.064
			MBE	0.0012	0.593	0.002	0.005	0.020	0.006	-0.006

*RBF* Radial basis function, *GRNN* Generalized regression neural network model, *RF* random forest, *SVR* Support vector regression, *R*^*2*^ Coefficient of determination, *RMSE* Root mean square error, *MBE* Mean bias error, *CAT* Catalase, *SOD* Superoxide dismutase, *APX* Ascorbate peroxidase, *POD* Peroxidase, *MDA* Malondialdehyde, and *H*_*2*_*O*_*2*_ hydrogen peroxide

### Optimization process 

The developed GRNN model, as the best ML algorithm with the highest prediction accuracy, was optimized using the NSGA-II algorithm to estimate the optimized level of inputs and finding the highest values of protein, APX, POD, CAT, SOD, and lowest values of MDA and H_2_O_2_ for the theoretical physio-biochemical traits of pomegranate fruits. The results of this multi-objective evolutionary search can be seen in Table 2. The optimization process by the GRNN-NSGA-II algorithm accurately predicted that the application of 20.90 mM of GABA treatment under drought-salinity stress conditions at 20.86 DPT would result in the maximum values of protein (0.80), APX (50.63), SOD (0.54), POD (1.53), and CAT (4.42) and minimum values of MDA (0.12), and H_2_O_2_ (0.44) for the ‘Atabaki’ cultivar, and that 20.54 mM of GABA treatment under drought-salinity stress after 20.72 DPT would result in the maximum values of protein (0.69), APX (51.51), SOD (0.53), POD (1.72), and CAT (5.66) and minimum values of MDA (0.15), and H_2_O_2_ (0.55) for the ‘Rabab’ cultivar.

**Table 2:** Multi-objective NSGA-II optimization of GRNN model to predict the best physio-biochemical parameters of pomegranate

Optimal level of independent variables				Optimal level of dependent variables						
**cultivar**	**Stress**	**GABA**	**DPT**	**Protein**	**APX**	**SOD**	**POD**	**CAT**	**MDA**	**H**_**2**_**O**_**2**_
‘Atabaki’	D × S	20.90	20.86	0.80	50.63	0.54	1.53	4.42	0.12	0.44
‘Rabab’	D × S	20.54	20.72	0.69	51.51	0.53	1.72	5.66	0.15	0.55

*DPT *Days post-treatment, *CAT* Catalase, *SOD* Superoxide dismutase, *APX* Ascorbate peroxidase, *POD* Peroxidase, *MDA* Malondialdehyde, and H_2_O_2 _Hydrogen peroxide

### Validation experiments 

Based on the results obtained from the validation experiment (Table 3), there was a negligible difference between the optimized-predicted results achieved from GRNN-NSGA-II and the experimental validation data for all of the pomegranate physio-biochemical responses. Indeed, based on the validation experiment, the predicted input variables using GRNN-NSGA-II resulted in 0.82 the protein, 60.21 the APX, 0.50 the SOD, 1.60 the POD, 5.25 the CAT, 0.08 the MDA, and 0.37 the H_2_O_2_ in the ‘Atabaki’ cultivar (Table 3). Also, based on the validation experiment, the predicted input variables using GRNN-NSGA-II resulted in 0.73 the protein, 49.25 the APX, 0.48 the SOD, 1.31 the POD, 5.12 the CAT, 0.10 the MDA, and 0.46 the H_2_O_2_ in the ‘Rabab’ cultivar (Table 3).

**Table 3:** Validation experiment of the predicted data through GRNN-NSGA-II for physio-biochemical traits of pomegranate

Treatment	Protein	APX	SOD	POD	CAT	MDA	H_2_O_2_
**Ideal point of NSGA-II process in ‘Atabaki’ cultivar**	0.82 ± 0.037	60.21 ± 2.393	0.50 ± 0.027	1.60 ± 0.387	5.25 ± 0.224	0.08 ± 0.004	0.37 ± 0.042
**Ideal point of NSGA-II process in ‘Rabab’ cultivar**	0.73 ± 0.056	49.25 ± 2.638	0.48 ± 0.031	1.31 ± 0.343	5.12 ± 0.322	0.10 ± 0.052	0.46 ± 0.043

Values in each column represent means ± SD. *CAT* Catalase, *SOD* Superoxide dismutase, *APX* Ascorbate peroxidase, *POD* Peroxidase, *MDA* Malondialdehyde, and H_2_O_2 _Hydrogen peroxide

## Discussion

Plants' primary defense responses to drought and salinity are very similar, as both conditions lead to reduced growth, photosynthesis, and stomatal aperture due to water stress. However, when plants face a combination of drought and salinity stress, their defensive reactions can differ from those observed under individual stress conditions. For instance, during prolonged periods of drought stress, root elongation occurs, and when plants are exposed to long-term salt stress, in addition to dehydration in plant organs, plants experience ionic stress and produce heavier roots with higher amounts of accumulated chloride, which in turn exerts an additional negative effect on plant growth. Therefore, metabolic responses to combined stress conditions are distinct and cannot be extrapolated from plant single stress responses [4]. The generation of ROS frequently causes membrane deterioration and organelle and cellular structural disintegration, and ultimately cell death [63]. ROS overproduction is associated with lipid peroxidation and the accumulation of MDA [17], which are considered biomarkers of oxidative stress caused by abiotic and biotic stresses [64]. H_2_O_2_, one of the extensively studied ROS, can oxidize proteins, lipids, and nucleic acids at high concentrations, rendering antioxidant enzymes and photosystems I and II inactive.

In general, the antioxidant enzymatic system is one of the plants' defense mechanisms to scavenge ROS caused by stress. Also, to adjust to drought and salinity-induced osmotic stress, plants accumulate compatible solutes or non-enzymatic secondary metabolites including proline, protein content, and soluble sugars [65, 66]. However, the efficacy of antioxidant enzymes in mitigating oxidative damage and their activity in response to stress depends on factors such as plant genotype/cultivar (resistant or sensitive), plant developmental stage, and severity and duration of drought or salinity stress [67]. In general, previous studies about the activity of antioxidant enzymes under salinity or water deficit conditions have revealed that the levels of antioxidant enzymes change differently, that is, they may increase, remain steady, or even decrease [68]. Our current findings demonstrated a significant increase in the content of H_2_O_2_, and consequently, MDA (oxidative stress parameters) under drought and salinity exposure stress. Interestingly, despite the increase in oxidative stress parameters, the activities of antioxidant enzymes (APX, SOD, POD, and CAT) and protein content also increased in the leaves of both cultivars under stress conditions. Hence, as the previous findings by researchers confirmed that there are different morphophysiological responses to abiotic stresses in pomegranate cultivars [22], we interpret these results as evidence that utilized cultivars are relatively resistant to water and salt stress. It is interesting to note that antioxidant enzyme activities and protein content in both cultivars increased markedly on the 30th DPT under stress treatment, whereas, at the end of the experimental period they showed a trend from ascent to descent. However, at the end of the experimental period, the activity of antioxidant enzymes and protein content was higher than the 14th DPT. Similar results in previous studies have been reported on the up-regulation of the antioxidant defense system to reduce injury from oxidative stress during drought or salinity conditions in pomegranate and various crops, such as black pepper [69], maize [70], and pistachio rootstocks [71]. In agreement with previous studies on other plant species under different environmental stress conditions [17, 19, 20], exogenous GABA supplied under drought-salinity stress conditions effectively enhanced the activities of antioxidant enzymes and reduced the production of ROS (H_2_O_2_) and MDA in pomegranate plants under drought-salinity stress conditions. This suggests that the ability of GABA-treated plants to regulate the osmotic balance and ROS scavenging in plant cells might be mainly due to the regulating activation of enzymatic metabolism during prolonged periods of drought and salinity stress. For instance, Abdel Razik et al. [20] reported the protective role of exogenous GABA in alleviating the oxidative stress induced by drought and heat stress and increasing the SOD, APX, and POD enzyme activities to control ROS in sunflower plants. Also, Wang et al. [19] demonstrated that GABA treatment helps to improve salt tolerance in maize seedlings by increasing the antioxidant enzyme systems such as SOD, CAT, APX, and POD and reducing the rate of MDA and superoxide anion (${{\text{O}}}_{2}^{\bullet -}$) content. Similarly, inhibition of H_2_O_2_ production, and oxidative damage to cell membranes through GABA application were observed in other crops subjected to drought and salt stress [2].

The influence of complex interactions between independent (input) variables on the physio-biochemical parameters of pomegranate cultivars in the current study is a complex and non-linear process, and analysis with traditional statistical techniques is insufficient for predicting exact the combination of inputs for the observed output variables. Therefore, we turned our attention to advanced technologies to gain insight into this mechanism. Computer-based software models have proven effective in predicting the outcome of various biological properties. In recent years, models based on ML have also been widely applied to identify and predict many other complex plant stress responses, such as disease resistance gene expression [72], transcription factor expression [73], crop yield [28, 74], morphological traits [33], and specialized metabolite biosynthesis [75]. However, previous studies primarily focused on evaluating individual models for modeling and predicting physio-biochemical plant studies [25, 76, 77], and a comprehensive comparison of different ML algorithms has not been conducted. In this study, we employed four ML algorithms (RBF, GRNN, RF, and SVM models) to model and estimate the results. To accurately evaluate the performance of these models, several performance metrics (R^2^, RMSE, and MBE) were considered for all ML algorithms. The results suggest that the GRNN model performed as robustly as the RBF, RF, and SVR models. However, overall, the GRNN technique demonstrated greater robustness than the other ML models in predicting pomegranate physio-biochemical traits. Previous findings in various plant biological sciences have strongly stated that GRNN is an accurate prediction model for modeling and prediction of results [51, 57, 78]. It is important to note that the input variables, output variables, and the specific model employed [23] influence the prediction capabilities of ML models. To interpret the results, NSGA-II was utilized to identify the key independent variables and predict the optimal combination of dependent variables. NSGA-II has been successful in various fields, including plant science [33, 79, 80]. In this study, the GRNN-NSGA-II method predicted that treatment with exogenous GABA at concentrations of 20.90 and 20.54 mM under drought-salinity stress, at 20.86 and 20.72 DPT, respectively, would maximize the activity of antioxidant enzymes and protein content, while minimizing the values of H_2_O_2_ and MDA traits in the ‘Atabaki’ and ‘Rabab’ pomegranate cultivars. Subsequently, the predicted results obtained from the developed method (GRNN-NSGA-II) were validated through experimental validation. These findings demonstrate that the mentioned advanced methodology is an effective approach for easier interpreting the results of GABA concentrations under drought and salinity stress, specifically in relation to the physiological and biochemical changes in pomegranate. In the future, the use of powerful computational tools with optimized techniques will provide new insights into monitoring complex environmental conditions and their interaction effects. The data derived from this study can serve as a basis for future research on estimating physio-biochemical responses to abiotic stresses in pomegranate plants. However, more comprehensive studies are required to clearly elucidate the molecular mechanism of antioxidant enzymes that are regulated by GABA exogenously. Future research should also explore the metabolic pathways affected by GABA and drought and the salinity and role of GABA as a stress signaling molecule, its influence on other physiological reactions against ROS such as gene and protein expression, and its effect on secondary metabolites and polyphenolic compounds. Incorporating a comprehensive experimental design that considers these variables is crucial for a thorough understanding of GABA's potential in enhancing soil drought-salinity tolerance of plants. It is important to note that the advantages of GABA in enhancing soil drought-salinity tolerance have not been well understood in previous research, and the potential impact of GABA on the physio-biochemical traits of pomegranate has not been explored.

## Conclusion

In this research, we used four different ML algorithms, namely the RBF, GRNN, RF, and SVR, for the first time, to estimate the changes in antioxidant enzymes activity, protein content, MDA, and H_2_O_2_ of pomegranate based on the effect of various GABA concentrations under drought and salinity stress and their interactions. Application of ML models showed that the GRNN model performed better than the other algorithms in terms of R^2^ and error measures for estimating results. Moreover, with the optimization method (GRNN-NSGA-II), we could interpret the results of different ranges of GABA concentration and drought and salinity stress effects on each cultivar more easily. The GRNN-NSGA-II model identified the best GABA concentration to reduce drought and salinity-induced damage in pomegranate. Based on the results of this study, exogenous GABA improved antioxidant enzyme systems and protein content, leading to a decrease of H_2_O_2_ and MDA content. Therefore, the knowledge from this study showed that the ML methods are reliable and easy tools for estimating the effect of exogenous GABA on the physio-biochemical traits of pomegranates under drought and salinity. Also, this strategy has great potential as an analytical method in applying stress monitoring on different crops on a large scale.

### Supplementary Information

**Additional file 1. **

## Data Availability

Data is provided within the manuscript or supplementary information files.
