# Supplementary material for: Predicting and optimizing reactive oxygen species metabolism in Punica granatum L. through machine learning: role of exogenous GABA on antioxidant enzyme activity under drought and salinity stress
Source: BMC Plant Biol. 2024 Jan 23;24:65. doi: 10.1186/s12870-024-04740-2 (PMC11293251; doi:10.1186/s12870-024-04740-2)
Supplement: Supplementary file 1 — Additional file 1. [file 12870_2024_4740_MOESM1_ESM.docx]

**Table S1**

The effect of GABA concentrations, days post-treatment (DPT), and cultivars subjected to salinity-drought stress on pomegranate physicochemical responses.

| Iutput variables | | | | Output variables | | | | | | |
| --- | --- | --- | --- | --- | --- | --- | --- | --- | --- | --- |
| cultivar | **Stress** | **GABA** | **DPT** | **Protein** | **APX** | **SOD** | **POD** | **CAT** | **MDA** | **H_2_O_2_** |
|  |  | (mM) |  | (mg g^-1^ FW) | (U g^-1^ FW) | (U g^-1^ FW) | (U g^-1^ FW) | (U g^-1^ FW) | (µmol g^-1^ FW) | (µmol g^-1^ FW) |
| ‘Atabaki’ | C | 0 | 14 | 0.59±0.025 | 29.74±1.554 | 0.39±0.008 | 0.55±0.041 | 2.95±0.266 | 0.032±0.005 | 0.67±0.064 |
|  | C | 0 | 30 | 0.58±0.006 | 25.34±1.180 | 0.52±0.005 | 0.74±0.013 | 1.53±0.420 | 0.076±0.006 | 0.49±0.149 |
|  | C | 0 | 45 | 0.78±0.005 | 31.13±2.456 | 0.36±0.013 | 0.72±0.032 | 3.31±0.431 | 0.077±0.006 | 0.44±0.037 |
|  | C | 10 | 14 | 0.63±0.022 | 31.77±3.036 | 0.39±0.008 | 0.56±0.021 | 2.95±0.251 | 0.059±0.009 | 0.83±0.075 |
|  | C | 10 | 30 | 0.64±0.006 | 26.84±1.046 | 0.53±0.005 | 0.77±0.050 | 1.85±0.066 | 0.072±0.004 | 0.55±0.145 |
|  | C | 10 | 45 | 0.69±0.036 | 30.60±1.773 | 0.35±0.018 | 0.77±0.040 | 4.01±0.534 | 0.072±0.010 | 0.45±0.033 |
|  | C | 20 | 14 | 0.63±0.061 | 31.01±3.409 | 0.40±0.014 | 0.57±0.010 | 3.35±0.523 | 0.043±0.029 | 0.92±0.143 |
|  | C | 20 | 30 | 0.64±0.010 | 28.17±1.502 | 0.53±0.005 | 0.84±0.018 | 3.47±0.249 | 0.067±0.006 | 0.62±0.039 |
|  | C | 20 | 45 | 0.68±0.003 | 32.09±2.082 | 0.38±0.017 | 0.79±0.021 | 4.39±0.163 | 0.069±0.007 | 0.48±0.031 |
|  | C | 40 | 14 | 0.62±0.022 | 32.23±1.739 | 0.40±0.005 | 0.61±0.024 | 3.69±0.190 | 0.067±0.007 | 1.06±0.010 |
|  | C | 40 | 30 | 0.63±0.007 | 31.73±2.487 | 0.53±0.010 | 0.85±0.024 | 3.74±0.107 | 0.069±0.006 | 0.40±0.036 |
|  | C | 40 | 45 | 0.68±0.026 | 31.27±1.837 | 0.40±0.010 | 0.84±0.022 | 4.66±0.062 | 0.072±0.008 | 0.48±0.021 |
|  | D | 0 | 14 | 0.69±0.026 | 31.53±2.030 | 0.40±0.008 | 0.67±0.026 | 3.64±0.129 | 0.184±0.017 | 1.01±0.062 |
|  | D | 0 | 30 | 0.62±0.010 | 35.61±1.024 | 0.53±0.014 | 0.84±0.029 | 3.73±0.117 | 0.171±0.006 | 0.69±0.097 |
|  | D | 0 | 45 | 0.72±0.039 | 45.62±1.394 | 0.41±0.013 | 0.75±0.026 | 4.83±0.148 | 0.115±0.009 | 0.72±0.022 |
|  | D | 10 | 14 | 0.64±0.057 | 30.38±1.087 | 0.40±0.013 | 0.68±0.017 | 3.84±0.160 | 0.165±0.003 | 0.89±0.122 |
|  | D | 10 | 30 | 0.69±0.013 | 36.36±1.073 | 0.54±0.013 | 0.86±0.010 | 3.83±0.074 | 0.145±0.009 | 0.60±0.087 |
|  | D | 10 | 45 | 0.68±0.022 | 55.55±1.093 | 0.41±0.010 | 0.76±0.010 | 5.31±0.154 | 0.092±0.010 | 0.67±0.021 |
|  | D | 20 | 14 | 0.71±0.053 | 34.20±3.029 | 0.42±0.010 | 0.71±0.022 | 3.82±0.193 | 0.158±0.003 | 0.88±0.124 |
|  | D | 20 | 30 | 0.70±0.021 | 36.26±1.749 | 0.55±0.008 | 0.85±0.032 | 3.86±0.123 | 0.138±0.012 | 0.61±0.050 |
|  | D | 20 | 45 | 0.72±0.022 | 61.91±0.831 | 0.42±0.006 | 0.78±0.013 | 5.25±0.235 | 0.087±0.008 | 0.63±0.048 |
|  | D | 40 | 14 | 0.73±0.042 | 37.74±2.835 | 0.40±0.028 | 0.74±0.016 | 4.20±0.189 | 0.151±0.003 | 0.86±0.109 |
|  | D | 40 | 30 | 0.70±0.015 | 37.72±0.829 | 0.56±0.014 | 0.88±0.017 | 4.24±0.172 | 0.143±0.010 | 0.58±0.104 |
|  | D | 40 | 45 | 0.76±0.019 | 64.44±0.785 | 0.43±0.010 | 0.83±0.029 | 6.46±0.327 | 0.088±0.009 | 0.62±0.034 |
|  | S | 0 | 14 | 0.65±0.044 | 38.20±1.432 | 0.40±0.013 | 0.61±0.013 | 3.85±0.160 | 0.210±0.019 | 1.59±0.193 |
|  | S | 0 | 30 | 0.68±0.018 | 30.99±1.491 | 0.53±0.010 | 0.77±0.022 | 3.63±0.140 | 0.137±0.003 | 0.49±0.024 |
|  | S | 0 | 45 | 0.69±0.032 | 47.30±0.782 | 0.42±0.008 | 0.77±0.006 | 4.32±0.157 | 0.108±0.004 | 0.62±0.070 |
|  | S | 10 | 14 | 0.68±0.013 | 41.34±1.253 | 0.39±0.017 | 0.70±0.013 | 3.75±0.095 | 0.136±0.012 | 0.85±0.056 |
|  | S | 10 | 30 | 0.67±0.019 | 32.19±1.168 | 0.55±0.006 | 0.82±0.024 | 3.79±0.041 | 0.108±0.015 | 0.49±0.072 |
|  | S | 10 | 45 | 0.71±0.010 | 47.20±0.719 | 0.44±0.006 | 0.79±0.013 | 4.55±0.128 | 0.105±0.003 | 0.58±0.035 |
|  | S | 20 | 14 | 0.69±0.015 | 42.81±0.472 | 0.40±0.010 | 0.74±0.021 | 3.82±0.106 | 0.148±0.016 | 0.80±0.063 |
|  | S | 20 | 30 | 0.66±0.024 | 41.84±0.723 | 0.56±0.005 | 0.92±0.022 | 3.89±0.078 | 0.122±0.005 | 0.50±0.077 |
|  | S | 20 | 45 | 0.73±0.011 | 50.26±0.703 | 0.45±0.014 | 0.81±0.022 | 4.71±0.224 | 0.098±0.011 | 0.49±0.013 |
| Iutput variables | | | | **Output variables** | | | | | | |
| cultivar | **Stress** | **GABA** | **DPT** | **Protein** | **APX** | **SOD** | **POD** | **CAT** | **MDA** | **H_2_O_2_** |
|  |  | (mM) |  | (mg g^-1^ FW) | (U g^-1^ FW) | (U g^-1^ FW) | (U g^-1^ FW) | (U g^-1^ FW) | (µmol g^-1^ FW) | (µmol g^-1^ FW) |
|  | S | 40 | 14 | 0.70±0.016 | 44.81±0.933 | 0.41±0.005 | 0.83±0.048 | 4.15±0.161 | 0.126±0.014 | 0.67±0.086 |
|  | S | 40 | 30 | 0.69±0.017 | 46.99±1.187 | 0.57±0.010 | 0.94±0.022 | 4.00±0.061 | 0.120±0.009 | 0.46±0.069 |
|  | S | 40 | 45 | 0.75±0.011 | 51.38±1.656 | 0.45±0.008 | 0.86±0.034 | 4.78±0.136 | 0.095±0.007 | 0.45±0.017 |
|  | D×S | 0 | 14 | 0.65±0.024 | 48.66±1.733 | 0.40±0.005 | 0.71±0.028 | 4.18±0.042 | 0.225±0.016 | 1.49±0.083 |
|  | D×S | 0 | 30 | 0.63±0.038 | 42.64±0.814 | 0.54±0.006 | 0.84±0.015 | 3.66±0.193 | 0.245±0.017 | 0.62±0.040 |
|  | D×S | 0 | 45 | 0.70±0.025 | 39.05±1.280 | 0.43±0.010 | 0.84±0.008 | 4.67±0.040 | 0.161±0.029 | 0.69±0.031 |
|  | D×S | 10 | 14 | 0.63±0.017 | 49.09±1.883 | 0.41±0.010 | 0.77±0.039 | 4.61±0.245 | 0.206±0.038 | 0.76±0.031 |
|  | D×S | 10 | 30 | 0.68±0.021 | 46.86±1.913 | 0.53±0.013 | 0.99±0.049 | 3.71±0.164 | 0.195±0.017 | 0.56±0.067 |
|  | D×S | 10 | 45 | 0.72±0.006 | 47.62±2.616 | 0.45±0.008 | 0.85±0.010 | 4.70±0.098 | 0.127±0.026 | 0.62±0.022 |
|  | D×S | 20 | 14 | 0.64±0.019 | 49.20±3.394 | 0.41±0.008 | 0.79±0.005 | 4.70±0.261 | 0.211±0.007 | 0.76±0.049 |
|  | D×S | 20 | 30 | 0.68±0.024 | 48.44±1.678 | 0.54±0.008 | 1.05±0.062 | 4.18±0.078 | 0.144±0.010 | 0.42±0.037 |
|  | D×S | 20 | 45 | 0.72±0.013 | 60.31±0.486 | 0.47±0.013 | 0.87±0.010 | 4.95±0.091 | 0.119±0.010 | 0.59±0.033 |
|  | D×S | 40 | 14 | 0.66±0.005 | 51.59±0.968 | 0.42±0.008 | 0.93±0.032 | 4.93±0.039 | 0.169±0.038 | 0.67±0.015 |
|  | D×S | 40 | 30 | 0.81±0.018 | 50.98±1.417 | 0.56±0.006 | 1.56±0.073 | 4.41±0.068 | 0.119±0.005 | 0.40±0.037 |
|  | D×S | 40 | 45 | 0.76±0.010 | 60.15±1.140 | 0.47±0.010 | 0.90±0.038 | 4.96±0.127 | 0.077±0.006 | 0.53±0.090 |
| ‘Rabab’ | C | 0 | 14 | 0.59±0.024 | 22.28±1.542 | 0.40±0.005 | 0.55±0.018 | 2.56±0.079 | 0.046±0.003 | 0.92±0.061 |
|  | C | 0 | 30 | 0.57±0.010 | 22.83±1.250 | 0.51±0.008 | 0.75±0.014 | 3.54±0.106 | 0.057±0.018 | 0.41±0.026 |
|  | C | 0 | 45 | 0.67±0.025 | 23.53±1.508 | 0.33±0.005 | 0.56±0.018 | 3.52±0.088 | 0.063±0.008 | 0.58±0.028 |
|  | C | 10 | 14 | 0.58±0.018 | 24.87±1.678 | 0.40±0.008 | 0.63±0.025 | 3.21±0.049 | 0.062±0.016 | 0.74±0.093 |
|  | C | 10 | 30 | 0.59±0.009 | 27.75±1.330 | 0.52±0.005 | 0.77±0.015 | 3.52±0.068 | 0.064±0.007 | 0.41±0.026 |
|  | C | 10 | 45 | 0.71±0.032 | 25.54±2.048 | 0.35±0.005 | 0.66±0.021 | 3.66±0.143 | 0.060±0.010 | 0.52±0.072 |
|  | C | 20 | 14 | 0.57±0.008 | 27.07±1.991 | 0.40±0.014 | 0.63±0.024 | 3.37±0.079 | 0.042±0.009 | 0.70±0.034 |
|  | C | 20 | 30 | 0.69±0.019 | 34.72±2.032 | 0.53±0.005 | 0.76±0.017 | 3.70±0.068 | 0.049±0.005 | 0.38±0.036 |
|  | C | 20 | 45 | 0.69±0.007 | 28.39±0.463 | 0.33±0.006 | 0.68±0.006 | 3.77±0.043 | 0.064±0.010 | 0.53±0.059 |
|  | C | 40 | 14 | 0.60±0.009 | 27.52±0.890 | 0.40±0.006 | 0.66±0.038 | 3.43±0.070 | 0.045±0.012 | 0.67±0.052 |
|  | C | 40 | 30 | 0.70±0.020 | 35.63±1.135 | 0.53±0.006 | 0.80±0.013 | 3.73±0.083 | 0.053±0.009 | 0.38±0.050 |
|  | C | 40 | 45 | 0.71±0.027 | 29.12±0.085 | 0.36±0.005 | 0.68±0.013 | 3.83±0.101 | 0.058±0.005 | 0.52±0.073 |
|  | D | 0 | 14 | 0.62±0.053 | 36.26±4.956 | 0.40±0.015 | 0.69±0.015 | 3.67±0.094 | 0.253±0.010 | 1.28±0.039 |
|  | D | 0 | 30 | 0.72±0.005 | 38.77±1.510 | 0.53±0.005 | 0.82±0.010 | 4.04±0.106 | 0.153±0.008 | 0.53±0.076 |
|  | D | 0 | 45 | 0.77±0.009 | 30.26±0.620 | 0.38±0.013 | 0.77±0.022 | 4.21±0.217 | 0.101±0.002 | 0.92±0.050 |
|  | D | 10 | 14 | 0.60±0.010 | 38.56±0.571 | 0.41±0.008 | 0.71±0.015 | 3.76±0.129 | 0.216±0.021 | 0.80±0.051 |
|  | D | 10 | 30 | 0.77±0.020 | 41.06±1.252 | 0.54±0.010 | 0.89±0.034 | 4.58±0.077 | 0.143±0.010 | 0.49±0.036 |
|  | D | 10 | 45 | 0.76±0.008 | 30.96±1.492 | 0.37±0.013 | 0.84±0.033 | 4.59±0.035 | 0.093±0.002 | 0.66±0.022 |
|  | D | 20 | 14 | 0.62±0.009 | 41.22±1.149 | 0.41±0.022 | 0.75±0.019 | 3.82±0.077 | 0.216±0.022 | 0.76±0.058 |
|  | D | 20 | 30 | 0.79±0.017 | 42.57±0.929 | 0.54±0.009 | 0.93±0.025 | 4.64±0.046 | 0.152±0.014 | 0.46±0.051 |
| Iutput variables | | | | **Output variables** | | | | | | |
| cultivar | **Stress** | **GABA** | **DPT** | **Protein** | **APX** | **SOD** | **POD** | **CAT** | **MDA** | **H_2_O_2_** |
|  |  | (mM) |  | (mg g^-1^ FW) | (U g^-1^ FW) | (U g^-1^ FW) | (U g^-1^ FW) | (U g^-1^ FW) | (µmol g^-1^ FW) | (µmol g^-1^ FW) |
| ‘Rabab’ | D | 20 | 45 | 0.76±0.015 | 35.99±0.617 | 0.39±0.008 | 0.87±0.021 | 4.73±0.067 | 0.089±0.003 | 0.55±0.062 |
|  | D | 40 | 14 | 0.64±0.014 | 51.73±0.923 | 0.42±0.010 | 0.75±0.021 | 3.89±0.129 | 0.161±0.040 | 0.73±0.041 |
|  | D | 40 | 30 | 0.80±0.010 | 44.20±0.377 | 0.54±0.008 | 0.94±0.017 | 4.78±0.120 | 0.128±0.025 | 0.49±0.024 |
|  | D | 40 | 45 | 0.80±0.018 | 37.53±1.041 | 0.40±0.008 | 0.88±0.010 | 4.80±0.168 | 0.078±0.002 | 0.45±0.024 |
|  | S | 0 | 14 | 0.62±0.029 | 38.37±1.891 | 0.41±0.008 | 0.65±0.008 | 3.58±0.066 | 0.187±0.012 | 0.77±0.034 |
|  | S | 0 | 30 | 0.74±0.011 | 39.47±0.908 | 0.53±0.008 | 0.81±0.026 | 4.17±0.112 | 0.155±0.007 | 0.58±0.054 |
|  | S | 0 | 45 | 0.77±0.011 | 48.51±0.869 | 0.39±0.017 | 0.82±0.017 | 4.35±0.050 | 0.097±0.003 | 0.57±0.021 |
|  | S | 10 | 14 | 0.61±0.021 | 39.27±1.347 | 0.43±0.010 | 0.72±0.017 | 3.64±0.071 | 0.197±0.023 | 0.72±0.029 |
|  | S | 10 | 30 | 0.75±0.013 | 40.00±1.493 | 0.53±0.015 | 0.84±0.013 | 4.64±0.098 | 0.137±0.019 | 0.50±0.041 |
|  | S | 10 | 45 | 0.75±0.012 | 49.50±0.695 | 0.38±0.010 | 0.86±0.010 | 4.53±0.101 | 0.087±0.004 | 0.54±0.029 |
|  | S | 20 | 14 | 0.66±0.027 | 41.50±1.529 | 0.45±0.010 | 0.74±0.029 | 3.95±0.104 | 0.149±0.017 | 0.72±0.041 |
|  | S | 20 | 30 | 0.74±0.012 | 41.97±0.584 | 0.53±0.013 | 0.88±0.013 | 4.74±0.138 | 0.134±0.008 | 0.43±0.052 |
|  | S | 20 | 45 | 0.77±0.008 | 50.78±1.804 | 0.40±0.005 | 0.90±0.017 | 4.66±0.043 | 0.086±0.004 | 0.54±0.074 |
|  | S | 40 | 14 | 0.68±0.010 | 43.48±0.974 | 0.46±0.013 | 0.77±0.026 | 4.13±0.186 | 0.116±0.021 | 0.64±0.025 |
|  | S | 40 | 30 | 0.75±0.008 | 43.06±2.757 | 0.54±0.032 | 0.92±0.017 | 4.66±0.097 | 0.135±0.009 | 0.47±0.006 |
|  | S | 40 | 45 | 0.79±0.009 | 51.86±1.426 | 0.41±0.008 | 0.94±0.013 | 4.78±0.120 | 0.078±0.003 | 0.47±0.037 |
|  | D×S | 0 | 14 | 0.62±0.007 | 47.93±0.300 | 0.41±0.010 | 0.67±0.010 | 4.43±0.126 | 0.224±0.015 | 1.46±0.175 |
|  | D×S | 0 | 30 | 0.67±0.020 | 44.82±1.161 | 0.52±0.005 | 1.45±0.056 | 4.39±0.068 | 0.166±0.032 | 0.76±0.040 |
|  | D×S | 0 | 45 | 0.75±0.010 | 79.33±1.482 | 0.39±0.010 | 1.46±0.074 | 4.69±0.243 | 0.128±0.004 | 0.61±0.043 |
|  | D×S | 10 | 14 | 0.63±0.033 | 50.03±1.256 | 0.41±0.005 | 0.67±0.024 | 4.64±0.188 | 0.207±0.008 | 1.13±0.056 |
|  | D×S | 10 | 30 | 0.67±0.052 | 46.20±0.890 | 0.52±0.008 | 1.45±0.115 | 4.57±0.223 | 0.148±0.018 | 0.61±0.045 |
|  | D×S | 10 | 45 | 0.68±0.011 | 83.61±1.706 | 0.40±0.013 | 1.70±0.095 | 4.80±0.211 | 0.112±0.012 | 0.59±0.034 |
|  | D×S | 20 | 14 | 0.64±0.028 | 54.18±2.598 | 0.41±0.005 | 0.71±0.013 | 4.73±0.089 | 0.195±0.026 | 1.02±0.078 |
|  | D×S | 20 | 30 | 0.68±0.021 | 49.33±0.992 | 0.53±0.005 | 1.65±0.106 | 4.73±0.224 | 0.163±0.013 | 0.56±0.064 |
|  | D×S | 20 | 45 | 0.78±0.005 | 85.42±1.709 | 0.40±0.005 | 1.70±0.025 | 4.97±0.156 | 0.127±0.005 | 0.54±0.040 |
|  | D×S | 40 | 14 | 0.64±0.022 | 55.57±2.940 | 0.43±0.010 | 0.72±0.022 | 5.55±0.321 | 0.178±0.022 | 1.01±0.045 |
|  | D×S | 40 | 30 | 0.69±0.017 | 50.52±2.024 | 0.54±0.006 | 1.80±0.032 | 5.70±0.067 | 0.132±0.022 | 0.52±0.024 |
|  | D×S | 40 | 45 | 0.77±0.017 | 92.14±4.607 | 0.42±0.024 | 1.77±0.190 | 5.62±0.067 | 0.094±0.011 | 0.55±0.022 |

Values in each column represent means ± SD. Control (C), drought (D), salinity (S), drought and salinity (D×S), catalase (CAT), superoxide dismutase (SOD), ascorbate peroxidase (APX), peroxidase (POD), and malondialdehyde (MDA), and hydrogen peroxide (H_2_O_2_).
